# A case of inguinal lymphaticovenular anastomosis for refractory lymphatic ascites after uterine cancer surgery

**DOI:** 10.1080/23320885.2024.2304617

**Published:** 2024-01-18

**Authors:** Atsuhiko Iwao, Noriko Ikari, Akihito Higashi, Yuki Moriuchi, Rina Akashi, Koumei Asabe, Kazuya Kashiyama, Yuriko Kitajima, Katsumi Tanaka

**Affiliations:** a Department of Plastic and Reconstructive Surgery, Nagasaki University Hospital, Nagasaki, Japan; b Department of Obstetrics and Gynecology, Nagasaki University Hospital, Nagasaki, Japan

**Keywords:** Inguinal lymphaticovenular anastomosis, refractory lymphatic ascites, uterine cancer, pelvic lympha­denectomy, para-aortic lymphadenectomy

## Abstract

We herein report a case of refractory lymphatic ascites after uterine cancer surgery treated with bilateral inguinal lymphaticovenular anastomosis (LVA). LVA was performed four months after the uterine cancer surgery in a patient with refractory ascites that had developed one month after the gynecologic surgery. One year and eight months after LVA, there was no recurrence of ascites accumulation.

## Introduction

Lymphatic ascites is a rare complication following lymph node dissection for gynecologic malignancies, with an incidence of approximately 4% [1]. Although therapeutic intervention is required in about half of lymphatic ascites cases [1], most cases resolve with conservative treatment, such as a low-fat, high-protein diet, or oral octreotide. On the other hand, some cases are refractory, and prolonged ascites can be fatal, causing malnutrition due to protein leakage and immunosuppression due to lymphocyte leakage. Such refractory cases require invasive treatment such as therapeutic lymphography [2], surgical ligation [3], or peritoneovenous shunting [4].

Lymphaticovenular anastomosis (LVA) was originally used as an effective surgical treatment for secondary lymphoedema of the extremities [5]. This surgical technique involves the microsurgical anastomosis of lymphatic vessels and small veins in the extremities and is intended to reduce lymphoedema by diverting the interrupted lymphatic flow to the venous system.

Recently, the usefulness of LVA for refractory ascites has been reported; however, most reports were performed in the lower extremities [6–8]. We herein present a case of refractory lymphatic ascites after uterine cancer surgery treated with LVA in the bilateral inguinal region.

## Presentation of case

A 70-year-old woman presented to a local hospital with abnormal uterine bleeding. Endometrial curettage revealed endometrioid adenocarcinoma, and contrast-enhanced magnetic resonance imaging showed tumor invasion into more than half of the myometrium. Positron emission tomography-computed tomography (CT) showed no distant metastases. The patient was diagnosed with uterine cancer and referred to the gynecology department of our hospital. Modified radical hysterectomy, bilateral salpingo-oophorectomy, omentectomy, pelvic lymphade­nectomy, and para-aortic lymphadenectomy were performed. The entire surgical procedure was performed in the open abdomen. Lymph node dissection was performed by ligation or surgical energy device cautery. The postoperative diagnosis was uterine cancer (FIGO stage IIIC1).

**Figure 1.**
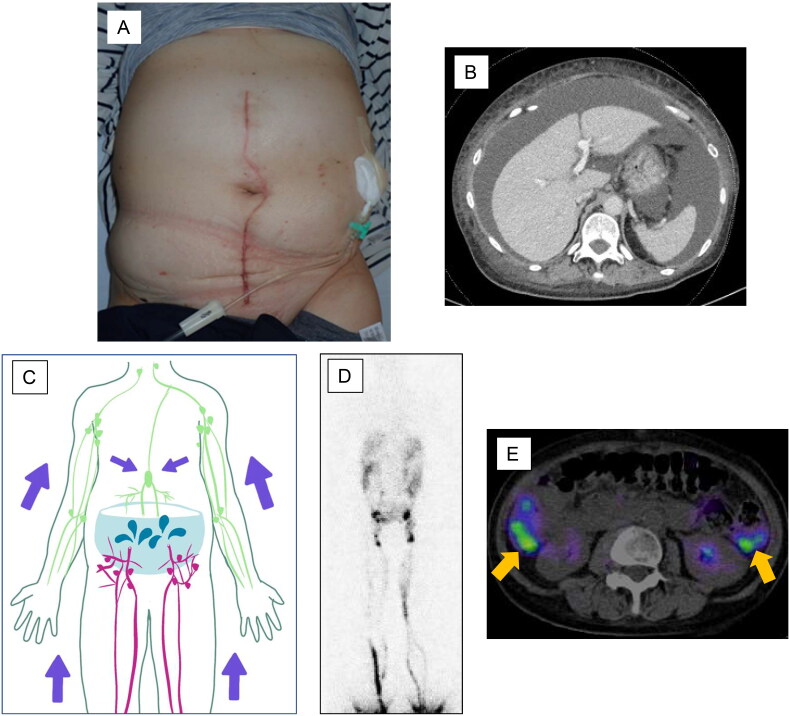
Preoperative findings. Abdominal distension is observed (A). Contrast-enhanced CT shows a large amount of ascites (B). The schema indicates that the lymphadenectomy resulted in a large amount of lymphatic fluid leakage and ascites accumulation (C). Lymphoscintigraphy or SPECT reveals leakage of the radioisotope into the peritoneal cavity. No secondary lymphedema is observed in either lower extremity (D). The arrow points to the leaked radioisotope (E).

**Figure 2.**
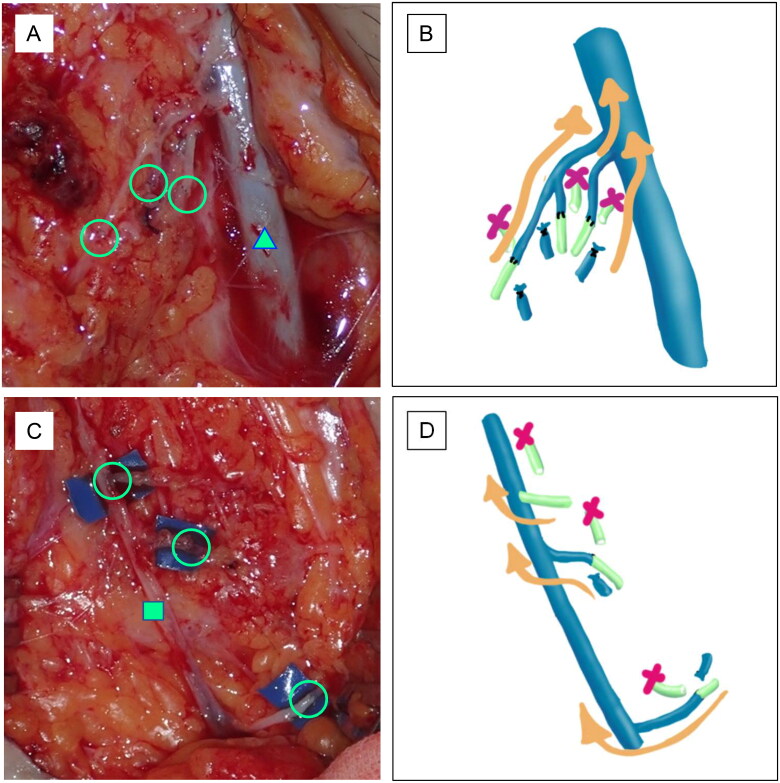
Intraoperative findings. Three lymphatic vessels and three veins are anastomosed in each of the right (A) and left (C) groin regions. The circles indicate the anastomoses. The triangle indicates the great saphenous vein (A). The tetragon indicates the accessory saphenous vein (C). The schema indicates the LVA on the right (B) and left (D) sides.

One month after surgery, the patient complained of dyspnea and abdominal distension (Figure 1(A)). Contrast-enhanced CT showed a large amount of ascites (Figure 1(B)). An abdominal puncture was performed and 2300 ml of ascites without chylous contamination was aspirated. Cytology showed no malignant cell contamination. It was thought that lymphatic leakage due to lymphadenectomy caused this accumulation of ascites (Figure 1(C)). Although approximately 3000 ml of ascites was aspirated once a week, the accumulation of ascites persisted. Therapeutic lymphography with Lipiodol was performed two months after surgery, but there was no improvement. Plastic surgeons were consulted. Lymphoscintigraphy with ^99m^Tc showed that the radioisotope reached the peritoneal cavity as early as 5 min after injection (Figure 1(D)), followed by findings of leakage of the radioisotope into the peritoneal cavity. There was no evidence of secondary lymphedema in either lower extremity. Single photon emission computed tomography (SPECT) also showed leakage of the radioisotope into the peritoneal cavity (Figure 1(E)). LVA was performed for refractory lymphatic ascites 4 months after surgery in the inguinal region. An approximately 10 cm incision was made in the bilateral inguinal region under general anesthesia based on the findings of indocyanine green lymphography. The superficial inguinal lymph node and great saphenous vein were identified in the right groin. The lymphatic vessels were dilated. Three afferent lymphatic vessels confluent with the lymph node were each anastomosed end-to-end with branches of the great saphenous vein (Figure 2(A,B)). The superficial inguinal lymph node and accessory saphenous vein were identified in the left groin region. The lymphatic vessels were slightly dilated. An afferent and an efferent lymphatic vessel confluent with the lymph node were each anastomosed end-to-end to a branch of the accessory saphenous vein. In addition, another afferent lymphatic vessel was anastomosed end-to-side to the main trunk of the accessory saphenous vein (Figure 2(C,D)). All anastomoses were performed with 11-0 nylon. The inflow of lymphatic fluid into the vein was observed. Plastic surgery performed the entire procedure.

**Figure 3.**
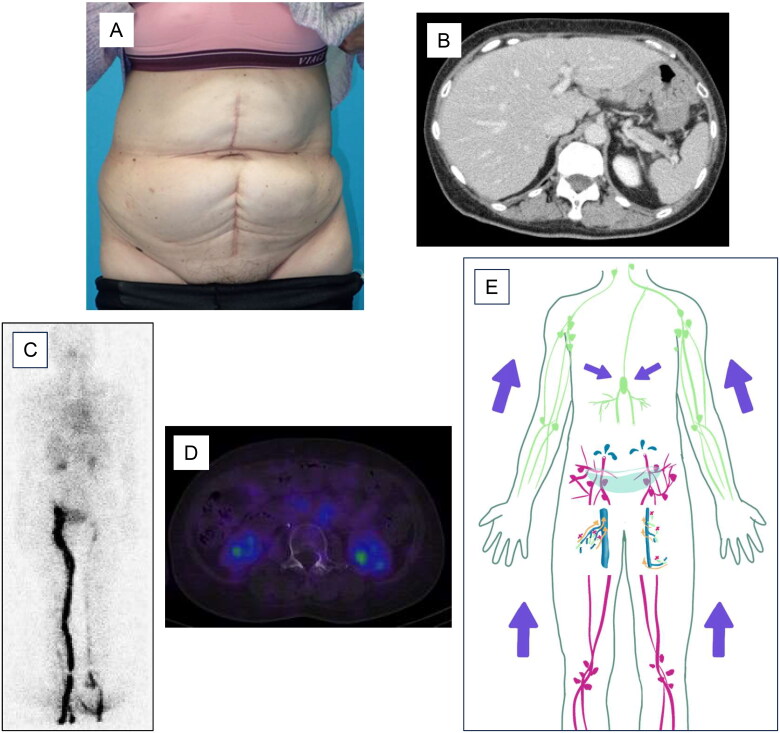
Postoperative findings. Abdominal distension disappears after LVA (A). Contrast-enhanced CT shows no evidence of ascites effusion (B). Lymphoscintigraphy (C) or SPECT (D) shows no evidence of radioisotope leakage into the peritoneal cavity. No secondary lymphedema is observed in either lower extremity. However, there is delayed lymphatic flow in the left lower extremity. The schema indicates that LVA reduced the amount of lymphatic fluid leaking into the abdominal cavity (E).

Three weeks after LVA, 3200 ml of ascites was aspirated once. Since then, no further accumulation of ascites has been observed. One year and two months after LVA, the abdominal distension had disappeared (Figure 3(A)) and contrast-enhanced CT showed no evidence of ascites effusion (Figure 3(B)). There was also no evidence of radioisotope leakage into the peritoneal cavity on lymphoscintigraphy (Figure 3(C)) or SPECT (Figure 3(D)). No secondary lymphedema was observed in either lower extremity. However, there was delayed lymphatic flow in the left lower extremity in lymphoscintigraphy. One year and eight months after LVA, there was no recurrence of ascites accumulation.

## Discussion

We successfully performed inguinal LVA for refractory ascites after uterine cancer surgery.

The lymphatic system in the abdominal cavity consists of the lumbar lymphatic trunk from the lower extremities or pelvic region, the hepatic lymphatic trunk, and the intestinal lymphatic trunk, which join the chyle cistern and then flow into the thoracic duct or right lymphatic trunk [9]. If the lymphatic vessels proximal to the chyle cistern or the intestinal lymphatic trunk itself are damaged, the leaking lymphatic fluid results in chyle ascites due to free fatty acids. Based on this lymphatic anatomy, our findings suggest that refractory lymphatic ascites caused by injury to the lumbar lymphatic trunk is a good indication for LVA in the lower extremity because LVA can reduce the excess lymphatic fluid entering the injured lymphatic vessels and contribute to the healing of these damaged lymphatic vessels [7] (Figure 3(E)). On the other hand, LVA may not always be effective in chyle ascites, where the lymphatic injury is suspected to be more proximal than in the chyle cistern, because the lymphatic fluid flowing into the injured area is not confined to the lower extremity [10]. The present case was lymphatic ascites. As the relatively large lymphatic vessel distal to the chyle cistern was damaged by lymph node dissection, LVA was effective.

In the present study, we performed inguinal LVA for refractory ascites. Most previous reports of LVA were performed in the thigh, lower leg, or foot, because the lymphatic vessels in the groin are large and the lymphatic vessels of the lower extremities converge in the inguinal region, allowing the inguinal LVA to efficiently return lymphatic fluid from the lower extremities to the veins. Lymphoscintigraphy showed a linear pattern with no dermal backflow in the lower extremity, and lymphatic flow in the lower extremity itself was normal. To the best of our knowledge, this is the first report of inguinal LVA for refractory ascites.

A previous study reported retroperitoneal LVA that was attempted for refractory chyle ascites [11]. The gonadal vein was transposed in the direction of the major chylous-leaking holes in the retroperitoneal region, and then these were anastomosed end to side. The amount of intra-abdominal drainage decreased from postoperative day 6, and the chyle ascites eventually disappeared. The method of performing LVA by directly approaching the site of lymphatic leakage is considered to be extremely effective in chyle ascites; however, laparotomy is invasive.

It remains controversial when to proceed with surgery in refractory ascites. Conservative treatment has a high response rate. However, we strongly recommend LVA in the groin and lower extremities for patients with prolonged treatment. Although the present case was performed under general anesthesia, LVA can be performed under local anesthesia [6,8] and is minimally invasive because it is performed in a shallower layer than the deep fascia.

## Conclusion

Inguinal LVA for refractory lymphatic ascites after pelvic and para-aortic lymphadenectomy is a useful technique because the lymph collected from the entire lower extremity is efficiently recirculated to the venous system. This case report suggests another treatment option for patients with refractory lymphatic ascites who are resistant to conservative treatment.

## Consent form

Written informed consent was obtained from the patient for publication of this case report and accompanying images.
